# Follow-up of late preterm infants: why, what and who?

**DOI:** 10.1186/1824-7288-40-S2-A26

**Published:** 2014-10-09

**Authors:** F Gallini, R Arena, V Romano, S Frezza, C Romagnoli

**Affiliations:** 1 Neonatal Intensive Care and Follow-up Unit, Department of Pediatrics, UCSC- Rome, Italy

## 

Late preterm infants (LPI) represent a growing population with own peculiar vulnerabilities; only recently attention has been focused on the impact of late preterm birth on child health, in order to define short and long-term outcomes [1,2].

LPI are physiologically and metabolically immature; they are at higher risk than term infants of developing medical complications, resulting in greater rate of mortality and morbidity not only in the neonatal period, but also during infancy, childhood, adolescence, and through adulthood [2,3].

Increasing evidence shows the association between late-preterm birth and various long-term medical and behavioral morbidities, including cerebral palsy, attention problems and antisocial behavior, as well as lower IQ, impaired cognitive and academic performance at school age. Fetal brain undergoes a dramatic growth and maturation during last four weeks of gestation, and this is probably the most important reason of LPI worse neurodevelopmental outcomes compared to full-term infants [3-5].

Physical development is an other important outcome for LPI; in addition to intrauterine growth restriction, LPI may be susceptible to feeding difficulty resulting in poor weight gain and underweight. Since failure to thrive in early infancy may be also associated with adverse cognitive and developmental outcomes, close monitoring of LPI growth pattern is needed [6].

Moreover, late-preterm birth has a negative effect also on maturation of the lungs, interrupting evolution from alveolar saccules to mature alveoli. LPI have been shown to develop early respiratory morbidities more frequently than infants born at term. However, the risk for long-term respiratory problems, such as asthma, has not yet been established in this group of patients [3,7].

Therefore LPI need a multidisciplinary, personalized and effective follow–up care that begins at birth and continues, with varying degrees of surveillance and reflecting individual needs, throughout the lifespan. Pediatricians must play a crucial role by ensuring that appropriate screening and assessments are completed, referrals are made and continuity of care is coordinated. They have to be aware of the major problems that LPI may encounter, providing anticipatory guidance when needed. Pediatricians together with parents, child development specialists, and education professionals need to know the possible school underachievement and behavioral problems so that prompt referrals to early intervention services are made [8,9].

Up to now, standardized short and long term follow-up schedule for LPI has not been developed yet; therefore, further research should focus on systematic evaluation of outcomes of LPI, in order to optimize follow-up monitoring of this population of children.
